# RhoA/ROCK Pathway Activation is Regulated by AT1 Receptor and Participates in Smooth Muscle Migration and Dedifferentiation via Promoting Actin Cytoskeleton Polymerization

**DOI:** 10.3390/ijms21155398

**Published:** 2020-07-29

**Authors:** Yan Qi, Xiuying Liang, Fan Dai, Haijing Guan, Jingwen Sun, Wenjuan Yao

**Affiliations:** Department of Pharmacology, School of Pharmacy, Nantong University, Nantong 226001, Jiangsu, China; 1923310019@stmail.ntu.edu.cn (Y.Q.); 1617042068@stmail.ntu.edu.cn (X.L.); 17170018@stmail.ntu.edu.cn (F.D.); 1627012011@stmail.ntu.edu.cn (H.G.); 1727011021@stmail.ntu.edu.cn (J.S.)

**Keywords:** angiotensin II, RhoA/ROCK, migration, dedifferentiation, actin

## Abstract

Background: In this study, we investigated the mechanism of Rho GTPases signaling on Ang II-mediated cell migration and dedifferentiation in human aortic vascular smooth muscle cells (HA-VSMCs) and an Ang II-infusion mouse model. Methods: Cells were pretreated with different inhibitors or Ang II. Cell migration was detected by Wound healing and Transwell assay. Mice were treated with Ad-RhoA-shRNA virus or Irbesartan or fasudil and then infused with Ang II. Results: Ang II treatment induced HA-VSMCs migration in a dose- and time-dependent manner and reduced the expression of VSMC contractile proteins. These effects were significantly suppressed by the inhibition of Ang II type 1 receptor (AT1 receptor), RhoA, and Rho-associated kinase (ROCK). Furthermore, Ang II treatment promoted the activation of RhoA and ROCK, which was reduced by AT1 receptor inhibition. Meanwhile, Ang II treatment induced F-actin polymerization, which was inhibited after ROCK inhibition. In mice, Ang II infusion increased VSMC migration into the neointima and reduced VSMC differentiation proteins levels, and these effects were shown to be dependent on AT1 receptor and RhoA/ROCK pathway. Conclusion: This study reveals a novel mechanism by which Ang II regulates RhoA/ROCK signaling and actin polymerization via AT1 receptor and then affects VSMC dedifferentiation.

## 1. Introduction

The main function of vascular smooth muscle cells (VSMCs) is contraction. VSMCs differentiation requires the expression of several contractile proteins; including smooth muscle α-actin (ACTA2), smooth muscle myosin heavy chain (MYH11), transgelin (TAGLN), and smoothelin [1]. In response to various pathological stimuli, VSMCs can be transited from a contractile phenotype to a proliferative synthetic phenotype; this transition or dedifferentiation is a key step in vascular remodeling and atherosclerotic lesion formation or other cardiovascular diseases [2]. VSMCs migration is one of the important manifestations of VSMCs synthetic phenotype. A general loss of critical VSMC contractile or differentiation markers including MYH11, SM22, and smoothelin occurred during smooth muscle dedifferentiation or phenotypic conversion [3,4,5]. There is considerable evidence implicating a role for angiotensin II (Ang II) in the inflammation, proliferation, migration, and hypertrophy process by activating signaling responses via its receptor [6,7,8]. Ang II exhibits growth and migration promoting effects in vascular smooth muscle cells (VSMCs), which contributes to vascular remodeling in cardiovascular disease under certain conditions [9,10,11]. Our recent research also indicates that Ang II affects HA-VSMC (human aortic vascular smooth muscle cell) proliferation and vascular remodeling via regulating RhoGDIs protein stability [12]. Unfortunately; the precise molecular mechanisms by which Ang II regulates VSMCs migration in human cell lines remain largely unclear.

The small Rho GTPase family of proteins, including the three major G-protein classes RhoA, Rac1, and Cdc42, have crucial roles in regulating multiple common cellular functions [13,14]. Rho-associated kinases (ROCK), one of the effector proteins of the Rho GTPases, phosphorylate a series of downstream targets and play crucial roles in various cellular functions such as cell contraction, migration and actin cytoskeleton organization [15,16,17]. Our previous studies also demonstrated that RhoA/ROCK pathway is responsible for TNF-α-mediated endothelial cytotoxicity and PDGF-BB-induced VSMC phenotypic modulation [2,18]. However, there is no evidence to explore whether Rho GTPases and its downstream signals are involved in Ang II-mediated cell migration or phenotype modulation in human aortic vascular smooth muscle cells (HA-VSMCs).

In the present study, we used an in vitro Ang II-mediated HA-VSMC dedifferentiation model and an in vivo Ang II-infusion vascular remodeling model to investigate the effects of Rho GTPases and its downstream pathways on HA-VSMC migration and phenotypic transformation.

## 2. Results

### 2.1. Ang II Promotes HA-VSMC Migration and Down-Regulates Differentiation Marker Proteins via AT1 Receptor

**Figure 1:**
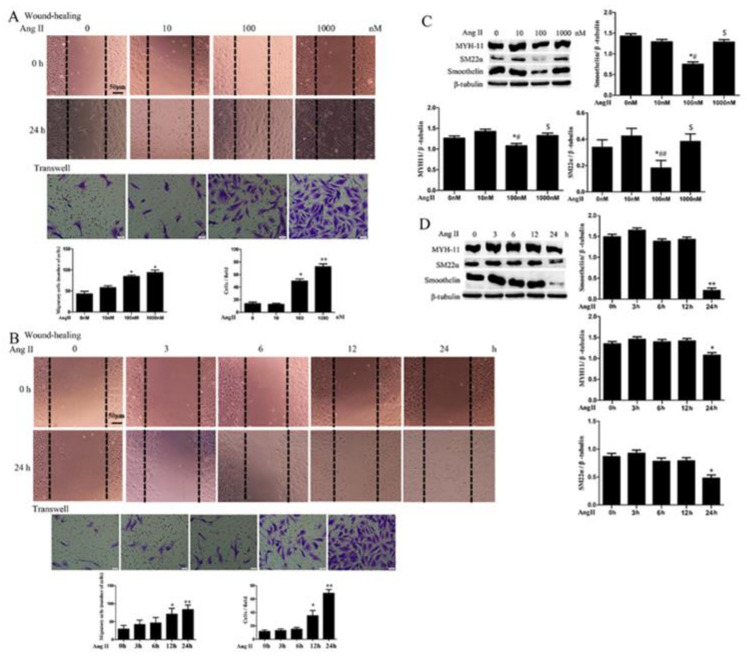
The effects of Ang II on cell migration and the expression of contractile marker proteins in human aortic vascular smooth muscle cells (HA-VSMCs). Cells were treated with Ang II at different concentrations (0, 10, 100, 1000 nM) or different time points (0, 3, 6, 12, 24 h). Untreated cells were used as the control. (**A** and **B**) Cell migration was detected by wound-healing and Transwell assays after Ang II treatment. Histograms show the quantification of the wound healing and Transwell assay results. The scar bar is 50 μm. * *p* < 0.05 and ** *p* < 0.01 vs. the control group (*n* = 3). (**C** and **D**) Western blot analysis of MYH11, SM22α, and smoothelin. Histograms show the ratios of myosin heavy chain (MYH11) or SM22α or smoothelin to β-tubulin. * *p* < 0.05 and ** *p* < 0.01 vs. the control group; # *p* < 0.05 and ## *p* < 0.01 vs. the group treated with 10 nM Ang II; $ *p* < 0.05 vs. the group treated with 100 nM Ang II (*n* = 3).

Ang II has been previously reported to activate VSMC migration in rat cells [19,20,21]. To investigate the effects of Ang II on HA-VSMCs dedifferentiation, we measured cell migration and the expression of contractile or differentiation marker proteins (MYH11, SM22α, smoothelin) after treatment with Ang II at different concentrations and time points in this study. As shown in Figure 1A, Ang II treatment for 24 h stimulated cell migration in a concentration-dependent manner in HA-VSMCs. In addition, 100 nM Ang II treatment promoted HA-VSMCs migration in a time-dependent manner (Figure 1B). However, Ang II treatment exerted different effects on the expression of contractile marker proteins in HA-VSMCs at different concentrations (Figure 1C). The expressions of MYH11, SM22α and smoothelin were significantly decreased at 100 nM of Ang II treatment when compared with the expressions at 0 and 10 nM. The expressions of these markers were subsequently increased at 1000 nM of treatment with Ang II when compared with the expressions at 100 nM. The result suggests that HA-VSMCs migration is not always consistent with the declined expression of contractile markers. When HA-VSMCs were treated with 1000 nM Ang II, it might be accompanied by changes or decreases of other differentiated proteins expression. Furthermore, the expressions of contractile markers were obviously decreased at 24 h of treatment with Ang II when compared with the control (Figure 1D).

**Figure 2:**
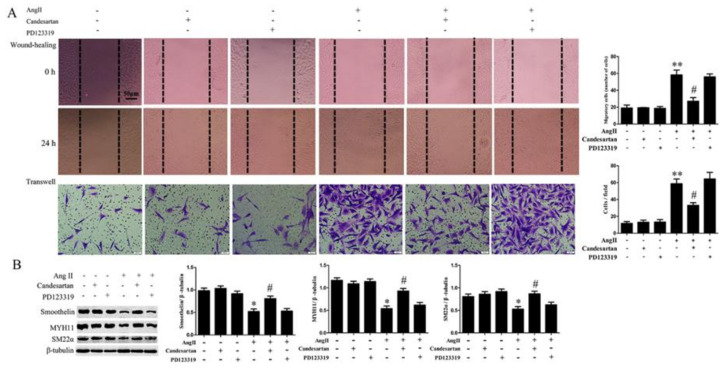
The effects of Ang II receptor inhibitors on cell migration and the expression of contractile marker proteins in HA-VSMCs. Cells were pretreated with candesartan (5 μM) for 6 h or PD123319 (5 μM) for 6 h and then exposed to 100 nM Ang II for 24 h. Untreated cells were used as the control. (**A**) Cell migration was detected by wound-healing and Transwell assays. Histograms show the quantification of the wound healing and Transwell assay results. The scar bar is 50 μm. ** *p* < 0.01 vs. the control group; # *p* < 0.05 vs. the Ang II-treated group (*n* = 3). (**B**) Western blot analysis of MYH11, SM22α, and smoothelin. Histograms show the ratios of MYH11 or SM22α or smoothelin to β-tubulin. * *p* < 0.05 vs. the control group; # *p* < 0.05 vs. the Ang II-treated group (*n* = 3).

To evaluate the role of AT1 and AT2 receptors on Ang II-mediated cell migration and dedifferentiation, we examined the effects of AT1 or AT2 receptor inhibitor on HA-VSMCs migration and the expressions of MYH11, SM22α, and smoothelin after 100 nM Ang II treatment for 24 h. As shown in Figure 2A, pretreatment with the AT1 receptor inhibitor candesartan obviously reduced Ang II-mediated cell migration in HA-VSMCs. In contrast, pretreatment with the AT2 receptor inhibitor PD123319 did not significantly affect HA-VSMCs migration at 24 h treatment with Ang II. Furthermore, candesartan pretreatment significantly promoted the expressions of MYH11, SM22α, and smoothelin in Ang II-treated HA-VSMCs (Figure 2B). Whereas, PD123319 did not affect the decline of contractile markers expression in HA-VSMCs treated with Ang II. These data suggest that Ang II regulates HA-VSMCs migration and dedifferentiation via AT1 receptor.

### 2.2. RhoA Activation Participates in HA-VSMC Migration and Dedifferentiation Induced by Ang II-AT1R Signaling

**Figure 3:**
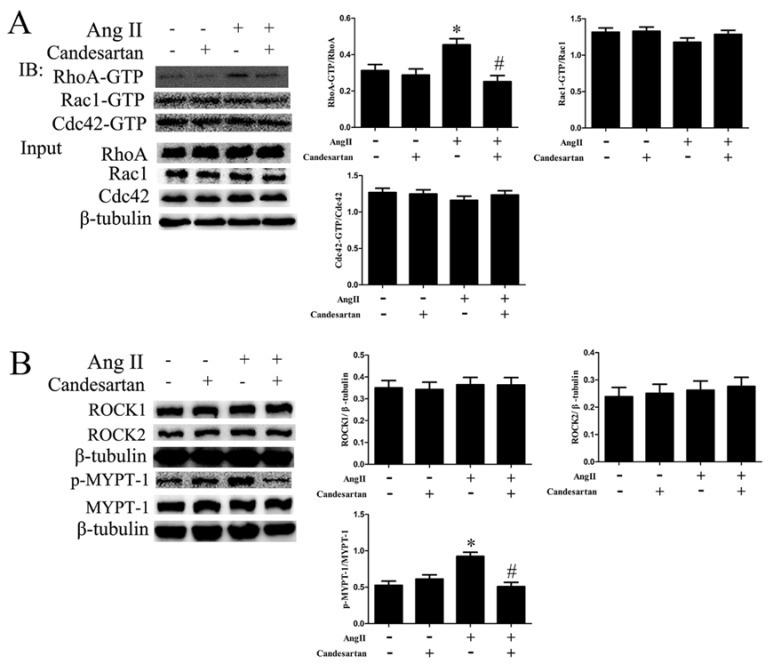
The effects of AT1 receptor inhibitor candesartan on the expression and activation of Rho GTPases and Rho-associated kinase (ROCK). Cells were pretreated with candesartan (5 μM) for 6 h and then exposed to 100 nM Ang II for 24 h. Untreated cells were used as the control. (**A**) Detection of Rho GTPases activity by pulldown assay. Histograms show the ratios of Rho GTPases GTP-bound to total Rho GTPases. * *p* < 0.05, vs. the control group; # *p* < 0.05 vs. the Ang II-treated group (*n* = 3). (**B**) Western blot analysis of the expression of ROCK1, ROCK2, MYPT1, and phospho-MYPT1 (Thr696). Histograms show the ratios of ROCK1 or ROCK2 to β-tubulin or phospho-MYPT1 to total MYPT1. * *p* < 0.05, vs. the control group; # *p* < 0.05 vs. the Ang II-treated group (*n* = 3).

**Figure 4:**
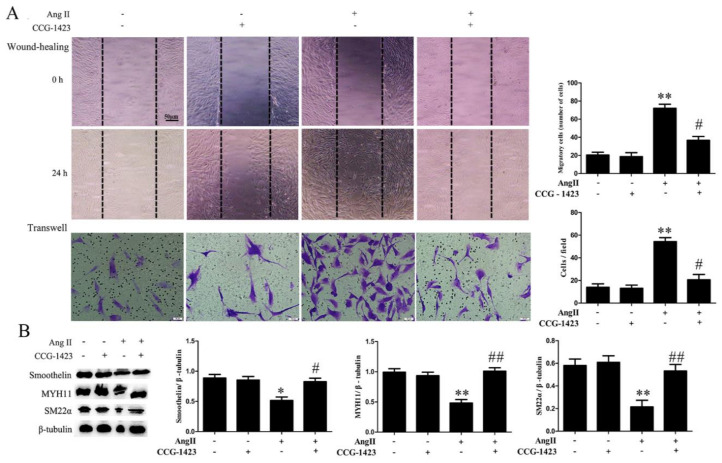
The effects of RhoA inhibitor CCG-1423 on cell migration and the expression of contractile marker proteins in HA-VSMCs. Cells were pretreated with CCG-1423 (20 μM) for 18 h and then exposed to 100 nM Ang II for 24 h. Untreated cells were used as the control. (**A**) Cell migration was detected by wound-healing and Transwell assays. Histograms show the quantification of the wound healing and Transwell assay results. The scar bar is 50 μm. ** *p* < 0.01 vs. the control group; # *p* < 0.05 vs. the Ang II-treated group (*n* = 3). (**B**) Western blot analysis of MYH11, SM22α, and smoothelin. Histograms show the ratios of MYH11 or SM22α or smoothelin to β-tubulin. * *p* < 0.05 and ** *p* < 0.01 vs. the control group; # *p* < 0.05 and ## *p* < 0.01 vs. the Ang II-treated group (*n* = 3).

**Figure 5:**
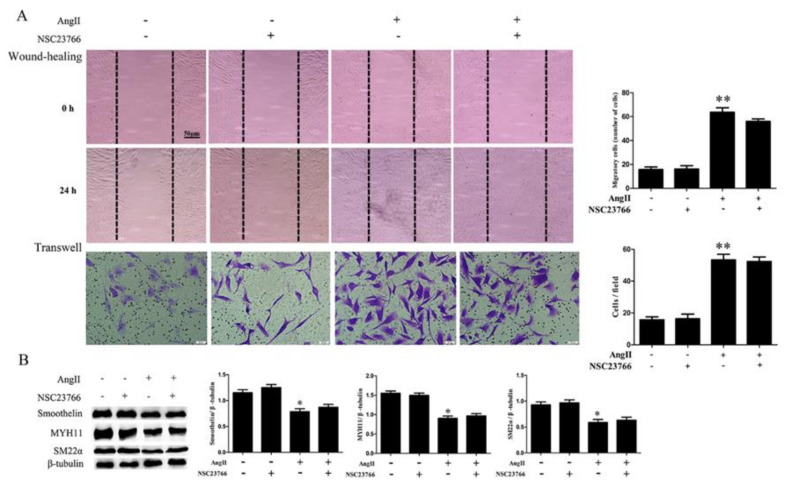
The effects of Rac1 inhibitor NSC23766 on cell migration and the expression of contractile marker proteins in HA-VSMCs. Cells were pretreated with NSC23766 (50 μM) for 30 min and then exposed to 100 nM Ang II for 24 h. Untreated cells were used as the control. (**A**) Cell migration was detected by wound-healing and Transwell assays. Histograms show the quantification of the wound healing and Transwell assay results. The scar bar is 50 μm. ** *p* < 0.01 vs. the control group (*n* = 3). (**B**) Western blot analysis of MYH11, SM22α, and smoothelin. Histograms show the ratios of MYH11 or SM22α or smoothelin to β-tubulin. * *p* < 0.05 vs. the control group (*n* = 3).

**Figure 6:**
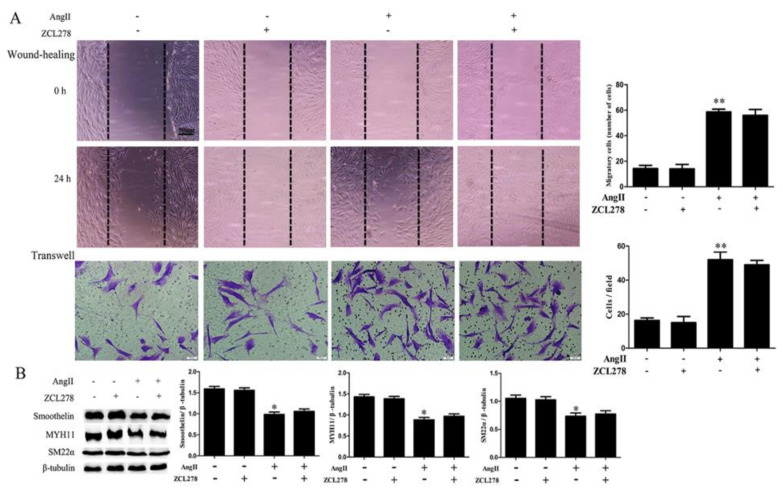
The effects of Cdc42 inhibitor ZCL278 on cell migration and the expression of contractile marker proteins in HA-VSMCs. Cells were pretreated with ZCL278 (50 μM) for 30 min and then exposed to 100 nM Ang II for 24 h. Untreated cells were used as the control. (**A**) Cell migration was detected by wound-healing and Transwell assays. Histograms show the quantification of the wound healing and Transwell assay results. The scar bar is 50 μm. ** *p* < 0.01 vs. the control group (*n* = 3). (**B**) Western blot analysis of MYH11, SM22α, and smoothelin. Histograms show the ratios of MYH11 or SM22α or smoothelin to β-tubulin. * *p* < 0.05 vs. the control group (*n* = 3).

To assess whether Rho GTPases participate in the regulation of Ang II on HA-VSMC phenotype transition, we analyzed the activation of Rho GTPases by pulldown assay. As shown in Figure 3A, there were no obvious increases in the active style of Rac1 or Cdc42 after Ang II treatment, while the active style of RhoA (RhoA GTP-bound) was significantly increased when cells were treated with Ang II. Candesartan pretreatment exhibited a significant inhibition on RhoA activation, but did not affect the activity of Rac1 or Cdc42 in Ang II-treated HA-VSMCs. In addition, RhoA inhibitor CCG-1423 pretreatment obviously decreased cell migration when compared with the Ang II-treated group (Figure 4A). Different from RhoA inhibition, both Rac1 and Cdc42 inhibition (NSC23766 and ZCL278 pretreatment) exhibited no significant inhibition on cell migration after Ang II treatment (Figure 5A and Figure 6A). Furthermore, we investigated the effects of Rho GTPases inhibition on the expression of VSMC differentiation markers in Ang II-treated HA-VSMCs. As illustrated in Figure 4B, the decline in the expression of MYH11, SM22α, and smoothelin was markedly reduced by RhoA inhibition in Ang II-stimulated HA-VSMCs. Nevertheless, in the inhibition of Rac1 and Cdc42, no significant changes on VSMC differentiation markers expression were observed in comparison to the Ang II-treated group (Figure 5B and Figure 6B). These data demonstrate that RhoA activation, but not Rac1 and Cdc42 activity, stimulated by Ang II-AT1 receptor signaling participates in HA-VSMC migration and dedifferentiation.

### 2.3. ROCK Activation Induced by Ang II-AT1R Signaling Participates in HA-VSMC Migration and Dedifferentiation by Promoting Actin Polymerization

**Figure 7:**
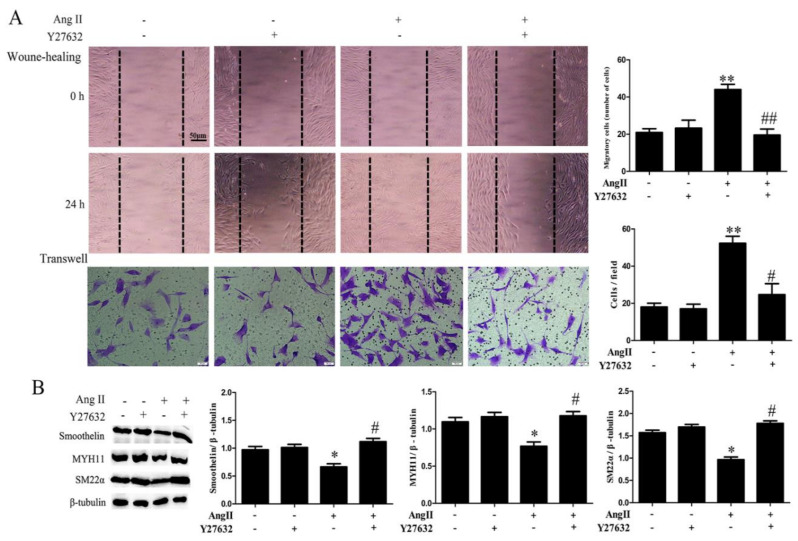
The effects of ROCK inhibitor Y27632 on cell migration and the expression of contractile marker proteins in HA-VSMCs. Cells were pretreated with Y27632 (20 μM) for 30 min and then exposed to 100 nM Ang II for 24 h. Untreated cells were used as the control. (**A**) Cell migration was detected by wound-healing and Transwell assays. Histograms show the quantification of the wound healing and Transwell assay results. The scar bar is 50 μm. ** *p* < 0.01 vs. the control group; # *p* < 0.05 and ## *p* < 0.01 vs. the Ang II-treated group (*n* = 3). (**B**) Western blot analysis of MYH11, SM22α, and smoothelin. Histograms show the ratios of MYH11 or SM22α or smoothelin to β-tubulin. * *p* < 0.05 vs. the control group; # *p* < 0.05 vs. the Ang II-treated group (*n* = 3).

To further clarify the effects of RhoA downstream kinase ROCK on Ang II-mediated migration and dedifferentiation, we analyzed the expression and activation of ROCK. The ROCK activity was detected by the phosphorylation of MYPT1 (myosin phosphatase target subunit 1) [22]. As shown in Figure 3B, Ang II exposure exhibited no significant effects on ROCK1 and ROCK2 expression. However, the activation of ROCK was significantly increased in compared to that of the control group, which was obviously reduced by AT1 receptor inhibition. Furthermore, we detected HA-VSMC migration and differentiation markers expression after ROCK inhibition. When cells were pretreated with ROCK inhibitor Y27632, cell migration induced by Ang II was dramatically decreased and the reduction of MYH11, SM22α and smoothelin expression was eventually improved (Figure 7).

**Figure 8:**
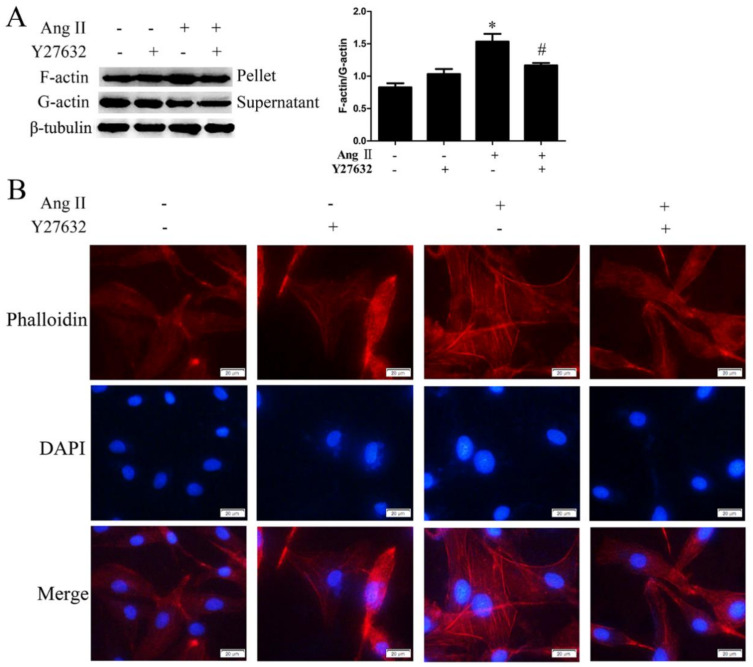
The effects of Y27632 pretreatment on F-actin polymerization in Ang II-treated HA-VSMCs. Cells were pretreated with Y27632 (20 μM) for 30 min and then exposed to 100 nM Ang II for 24 h. Untreated cells were used as the control. (**A**) The ratio of F-actin to G-actin analyzed by G-actin/F-actin In Vivo Assay Kit and Western blot analysis. Histogram shows the ratio of F-actin to G-actin. * *p* < 0.05 vs. the control group; # *p* < 0.05 vs. the Ang II-treated group (*n* = 3). (**B**) Phalloidin conjugates for staining F-actin filaments. Immunofluorescence assay for F-actin (red) and nuclei stained with DAPI (blue). The scar bar is 20 μm.

It has been confirmed that ROCK participates in cell detachment and apoptosis by regulating actin cytoskeleton reorganization [15]. However, the regulatory role of ROCK on actin polymerization in Ang II-mediated cell migration and dedifferentiation remains unclear. In this study, we observed the effects of Y27632 on actin polymerization and depolymerization by detecting the protein ratio of F-actin to G-actin. As shown in Figure 8A, cells treated with Ang II expressed significantly higher levels of F-actin/G-actin ratio compared with the control group, and this effect was inhibited by Y27632 treatment. This suggests that Ang II promotes actin polymerization by activating ROCK. Furthermore, we observed the effects of Y27632 on F-actin skeleton morphology by performing phalloidin staining. As shown in Figure 8B, F-actin stained as red filaments were evenly distributed in the cytoplasm in the control and Y27632 pretreatment group. After treatment with Ang II, red fluorescence and filamentous F-actin were significantly enhanced, and were mainly distributed at the cell edge. This effect was inhibited by pretreatment with Y27632, as showing the decreased filamentous F-actin at the cell edge. This result further illustrates that the polymerization of F-actin depends on ROCK activity.

### 2.4. RhoA/ROCK Pathway Participates in Ang II-Mediated VSMC Migration and Dedifferentiation in Mice

**Figure 9:**
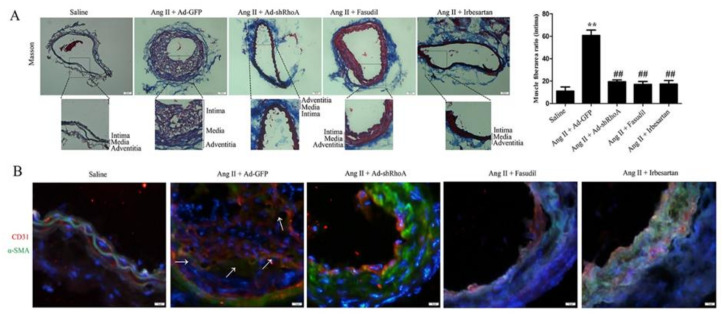
The effects of RhoA/ROCK pathway suppression or AT1 receptor inhibition on VSMC migration in Ang II-infused mice. Ang II was subcutaneously infused at a rate of 1000 ng/kg/min using the Alzet Model 1002 osmotic minipump. The group infused with normal saline was used as the control. The right common carotid arteries of mice were injected and incubated with 1 ×10 ^10^ pfu virus solution specific for RhoA knockdown for 20 min. The AT1 receptor inhibitor Irbesartan (50 mg/kg/d) and ROCK inhibitor fasudil (30 mg/kg/d) were added to drinking water for consumption by mice. (**A**) Masson’s trichrome staining of carotid arteries 14 days after Ang II infusion. Masson’s trichrome staining shows the collagen fibers (blue) and muscle fibers (red). We marked the location of the intima, media, and adventitia. Histogram shows the ratio of muscle fiber area in the intima. The scar bar is 50 μm. ** *p* < 0.01 vs. the saline-infused group; ## *p* < 0.01 vs. the model group (Ang II infusion + Ad-GFP virus) (*n* = 10). (**B**) Immunofluorescence assay for α-SMA (green) and CD31 (red) as double staining (merge). Nuclei were stained with DAPI (blue). White arrows display that Ang II promotes the expression of α-SMA in the neointima, while α-SMA expression remains in the media in the control and other groups. The scar bar is 20 μm.

**Figure 10:**
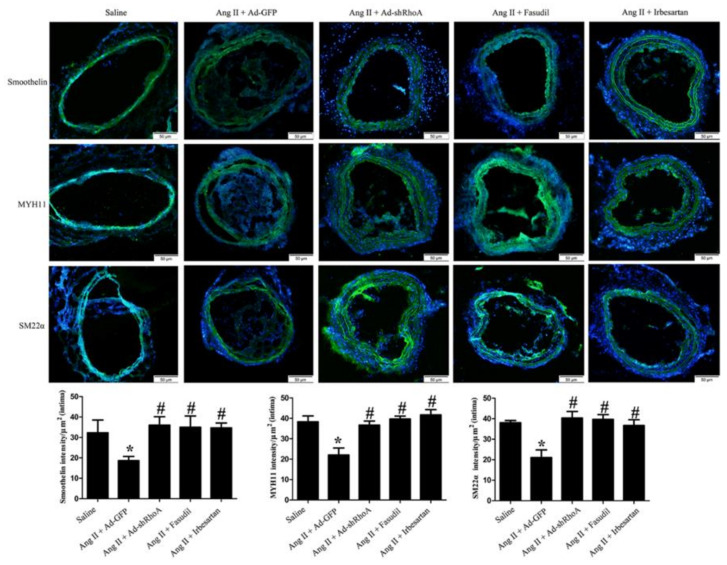
Immunofluorescence staining of MYH11, SM22α, and smoothelin (green). Nuclei were stained with DAPI (blue). Mice were treated the same way as in tablee 7. Histograms show the fluorescence intensity of the staining in the intima. * *p* < 0.05 vs. the saline-infused group; # *p* < 0.05 vs. the model group (Ang II infusion + Ad-GFP virus) (*n* = 10). The scar bar is 50 μm.

We next verified the effects of RhoA and ROCK activation on VSMC migration and dedifferentiation in vivo using a mouse Ang II infusion model. To investigate whether muscle fibers migrate into the neointima, we firstly examined myofiber production by Masson’s trichrome staining. As shown in Figure 9A, the ratio of muscle fiberarea (red staining) in neointima was significantly increased after Ang II infusion, and this effect was inhibited by RhoA knockdown or Fasudil or Irbesartan treatment. After RhoA/ROCK signaling suppression or Irbesartan treatment, the muscle fibers were retained in the media in mice. Secondly, we detected the expression of CD31 (endothelial marker) and α-SMA (myofiber marker) using immunostaining assay. As shown in Figure 9B, Ang II infusion resulted in significant α-SMA expression and co-localization of α-SMA with CD31 in neointima, suggesting that VSMC migrates into the neointima in Ang II-infused mice. Knockdown of RhoA and Fasudil or Irbesartan treatment reduced the co-localization of α-SMA with CD31 in the intima after Ang II infusion in mice, which confirms that RhoA/ROCK pathway is required for Ang II-mediated migration of VSMCs. Immunostaining assay also showed that the expression of MYH11, SM22α and smoothelin in neointima was significantly decreased after Ang II infusion (Figure 10), and these effects were inhibited by RhoA knockdown or Fasudil or Irbesartan treatment. RhoA/ROCK signaling suppression or inhibition promoted the expression of VSMC differentiation markers, which suggests that RhoA/ROCK pathway plays a key role in VSMC dedifferentiation induced by Ang II. These results are consistent with our in vitro results with HA-VSMCs. Overall, these results suggest that RhoA/ROCK signaling participates in Ang II-induced myofiber migration and dedifferentiation via AT1 receptor.

## 3. Discussion

Adult VSMCs play key roles in regulating blood vessel contraction and express contractile marker genes [1]. It is noted that VSMCs switch from the contractile phenotype toward proliferative, migratory, and/or inflammatory phenotypes under the presence of PDGF-BB [23], insulin-like growth factors (IGFs) [3], interferon gamma (IFN-γ) [24], Ang II [19,20,25] or in spontaneously hypertensive rats (SHR) [26]. It has been reported that the RhoA pathway plays an important role in Ang II-induced VSMC remodeling [25], stroke-prone spontaneously hypertension [27], cardiac remodeling [28,29], and vasoconstriction [30,31] in rats, mice or beagles. However, the effects of different Rho GTPase members on Ang II-mediated smooth muscle migration and dedifferentiation in human cell lines and the mechanisms behind these effects are not well understood, though we recently revealed a novel mechanism by which Ang II regulates VSMC proliferation by Rho-specific guanine nucleotide dissociation inhibitor (RhoGDI) protein stability [12]. In this study, for the first time, we demonstrated that RhoA activity, rather than Rac1 or Cdc42 activity, is regulated by AT1 receptor and participates in Ang II-induced HA-VSMC migration and dedifferentiation.

It has been reported that Ang II promotes cell migration in rat VSMCs [19]. However, the effects of Ang II on human VSMC migration and contractile genes expression have not been elucidated. VSMC phenotypic modulation is coupled to both promotion of migration or proliferation and down-regulation of VSMC differentiation marker genes [32]. In this study, we demonstrated that human VSMC migratory phenotype induced by Ang II is not completely consistent with the decreased expression of differentiation marker proteins. Ang II promoted human VSMC migration in a dose- and time-dependent manner. However, the expression of contractile proteins was significantly decreased after 100 nM Ang II treatment for 24 h, and was then increased until 1000 nM of Ang II treatment in HA-VSMCs. The possible reason is that when the concentration of Ang II treatment is further increased, VSMC protects the expression of contractile proteins through an unknown mechanism. But the migratory pathways are still activated, thus HA-VSMC maintains the migration phenotype. In the following experiments, we chose 100 nM Ang II treatment for 24 h as the condition for HA-VSMC migration and dedifferentiation.

In addition, in this study we demonstrated that Ang II regulated HA-VSMC migration and dedifferentiation and promoted the activation of RhoA and ROCK via AT1 receptor, although it had no significant effect on the activation of Rac1 and Cdc42 and the expression of ROCK1 and ROCK2. This result suggests that Ang II may promote HA-VSMC dedifferentiation through the RhoA-ROCK pathway without association with other Rho GTPases and their downstream signals. Also, these pathophysiological actions of Ang II are mediated by the activation of its best-characterized receptor: AT1 receptor. To further confirm the roles of Rho GTPases in Ang II-induced cell migration and dedifferentiation, we used different Rho GTPases inhibitors. Both RhoA inhibitor and ROCK inhibitor significantly alleviated HA-VSMC migration and promoted contractile proteins expression after Ang II treatment. Meanwhile, Rac1 inhibitor and Cdc42 inhibitor exerted no significant effects on Ang II-induced HA-VSMC migration and dedifferentiation. These data further demonstrate that RhoA and its downstream ROCK play important roles in human smooth muscle dedifferentiation.

It has been reported that RhoA/ROCK signaling pathway regulated actin cytoskeleton and subsequently regulated cell function [33,34,35]. ROCK inhibition participates in actin cytoskeleton rearrangement and thus allows for induction of a neural phenotype in placental-derived multipotent cells (PDMCs) and reduction of cell detachment and apoptosis [15,36]. Understanding the mechanisms that directly or indirectly regulate the actin cytoskeleton is an important area of research and quantitation of the F-actin/G-actin ratio is a useful metric in helping define these mechanisms. In the current study, we demonstrated that Ang II promoted the F-actin/G-actin ratio and F-actin polymerization, which was inhibited by ROCK inhibition. Therefore, we speculate that Ang II regulates F-actin polymerization via RhoA/ROCK pathway and then promotes HA-VSMC migration and dedifferentiation.

We also demonstrated that RhoA, ROCK, and AT1 receptor participate in VSMC migration from the media to neointima in mice after Ang II infusion; these effects were significantly inhibited by RhoA knockdown and Fasudil or Irbesartan treatment. Moreover, RhoA/ROCK suppression and AT1 receptor inhibition dramatically increased the expression of contractile marker proteins in Ang II-infused mice. Consistent with the in vitro results, these findings suggest that Ang II induces RhoA/ROCK signaling activation via the AT1 receptor, which plays a role in HA-VSMC migration and dedifferentiation. In summary, the main findings in this study are: (1) The RhoA/ROCK signaling pathway is involved in regulating Ang II-induced smooth muscle migration and dedifferentiation; (2) AT1 receptor participates in Ang II-mediated RhoA/ROCK pathway activation; (3) ROCK plays an important role in Ang II-induced F-actin polymerization.

## 4. Materials and Methods

### 4.1. Materials

Ang II was obtained from MedChemExpress (#HY-B0202; State of New Jersey, NJ, USA). G-actin/F-actin In Vivo Assay Kit (# BK037) and Rac1/Rho/Cdc42 GTPase activity assay kit (# BK030) were purchased from Cytoskeleton, Inc (Denver, CO, USA). Candesartan (AT1 receptor inhibitor; #139481–59–7), PD123319 (AT2 receptor inhibitor; #130663–39–7), CCG-1423 (RhoA inhibitor; # 285986–88–1), NSC23766 (Rac1 inhibitor; # 587841–73–4), ZCL278 (Cdc42 inhibitor; # 1177865–17–6) were from ChemCatch Co., Ltd. (Shanghai, China). Y27632 (ROCK inhibitor) was sourced from Selleckchem (#129830–38–2; Houston, TX, USA). Irbesartan (#HY-B0202) was purchased from MedChemExpress (NJ, USA). Fasudil was purchased from Yuan Ye Biological Technology Co., Ltd. (#105628–07–7; Shanghai, China). MMC (mitomycin C) was from Solarbio (#YZ-1444707; shanghai, China). Transwells (8.0 mm pore size) were purchased from Corning Incorporated (#3422; Corning, NY, USA). Crystal violet was purchased from Beyotime Technology (# C0121; Shanghai, China). Primary antibodies against smoothelin (# 6745), MYH11 (# 10827), SM22α (# A6760) were from ABclonal (Wuhan, China). Primary antibody against β-tubulin (# AT0003) was purchased from CMCTAG Inc (California, CA, USA). Anti-ROCK1 (ab45171), -ROCK2 (ab125025), -CD31 (ab24590) and -α-SMA (ab134964) antibodies were from Abcam (Cambridge, MA, USA). Anti-RhoA (# AF2179) and -Cdc42 (# AF2794) antibodies were from Beyotime Technology (Shanghai, China). Anti-MYPT1 (# 22117–1-AP) and -Rac-1 (# 24072–1-AP) antibodies were obtained from Proteintech (Chicago, IL, USA). Anti-phospho-MYPT1 (Thr-696) (sc-17556) antibody was from Santa Cruz Biotechnology (Santa Cruz, CA, USA). Peroxidase-conjugated AffiniPure Goat Anti-Rabbit IgG (H + L) was from Proteintech (SA00001–2; Chicago, IL, USA). FITC-conjugated Goat Anti-Rabbit IgG (H + L) was from ABclonal (# AS011; Wuhan, China). Cy3-conjugated AffiniPure Goat Anti-Mouse IgG (H + L) was obtained from Proteintech (# SA-00009–1; Chicago, IL, USA). Masson’s trichrome stain kits (G1340) were from Solarbio Life Sciences (Beijing, China). The BCA Protein Assay Kit was obtained from CWBiotech (CW0014S; Beijing, China). All other chemicals used in this study were analytical grade and were made in China.

### 4.2. Cell Culture

Human aortic vascular smooth muscle cells (HA-VSMCs) were purchased from American Type Culture Collection (ATCC number: CRL-1999; Manassas, VA, USA). The culture method refers to our previous published papers [12]. Cells were exposed to Ang II (100nM) at different time points (0, 3, 6, 12, and 24 h) or treated for 24 h with different concentrations of Ang II (0, 10, 100, and 1000 nM). Prior to Ang II treatment (100 nM, 24 h), HA-VSMCs were pretreated with candesartan (5 μM) or PD123319 (5 μM) for 6h; Y27632 (20 μM) for 30min; CCG-1423 (20 μM) for 18 h; NSC23766 (50 μM) for 30 min; ZCL278 (50 μM) for 30 min.

### 4.3. Wound Healing Assay

The method refers to our previous published papers [2]. Briefly, cells were cultured in the medium in the absence of FBS for 24 h before a sterile pipette tip was used to wound the monolayer cells. Subsequently, the cells were treated with 10 μg/mL MMC for 1 h before Ang II (100 nM) induction for 24 h. Numbers of migratory cells from the scratched boundary were counted and averaged from the resulting four phase images for each point with a light microscope (Olympus, Japan). The data were generated from three independent experiments.

### 4.4. Transwell

The method refers to our previous published papers [2]. Briefly, cells suspending in serum-free media added into the upper chamber. Cells were also pretreated with 10 μg/mL MMC for 1 h to prevent HA-VSMC proliferation. Cell migration was induced by 100 nM Ang II to the lower chamber with the medium containing 10% FBS. Cells were allowed to migrate for 24 h and nonmigrated cells in the upper chamber were removed with a cotton swab. Migrated cells on the lower side of the membrane and the lower chamber were fixed with paraformaldehyde and stained with crystal violet. The stained cells were counted in five randomly selected fields per well using an inverted microscope.

### 4.5. Western Blotting

Cells with various treatments were lysed in protein lysis buffer (1% NP-40, 1mM PMSF). Then, the lysates were centrifuged at 4 °C for 15 min at 12,000 r/min, and the protein concentrations were determined using Bradford assays. To isolate filamentous actin (F-actin) and globular/monomer actin (G-actin), a G-actin/F-actin In Vivo Assay Kit was used according to the manufacturer’s instructions. Subsequently, the protein samples were separated using SDS-polyacrylamide gel electrophoresis (PAGE) and transferred to nitrocellulose membranes. The membranes were then blocked with 5% fat-free milk for 2 h at room temperature and incubated with primary antibodies overnight at 4 °C. The blots were detected by HRP-conjugated secondary antibodies for 2 h at room temperature followed by analysis with an ECL detection system (Amersham Biosciences). β-tubulin was used as an internal standard. The relative intensities of the signals were quantified using densitometry and Imaging software (Labworks).

### 4.6. Pulldown Assay

Cells were seeded at 5 × 10^4^ cells per ml and grown for 3–5 days. Serum starvation or other treatment should be performed when cells are approximately 30% confluent. Then, the culture dish was retrieved from incubator and immediately aspirated out all of the media and placed firmly on ice. Immediately, the cells were rinsed with an appropriate volume of ice cold PBS to remove serum proteins and then incubated with lysis buffer containing 1× protease inhibitor cocktail on ice. A sample of at least 20 µL should be kept on ice for protein concentration measurement. A 20–50 µg aliquot of each sample should also be kept for Western blot quantitation of total RhoA, Rac1, or Cdc42. The total cell protein of 500 μg was added to a 1.5 mL Ep tube containing 30 µL of rhotekin-RBD beads (for RhoA activation assay) or 10 μL of PAK-PBD beads (for Rac1 and Cdc42 activation assays) prepared in advance. The beads and protein lysates were incubated at 4 °C on a rotator or rocker for 1 h and then centrifuged at 4 °C for 1 min. The supernatant was removed and the binding beads were washed using 500 μL of wash buffer each time in a manner that completely resuspends the beads. The beads binding the active Rho GTPases were centrifuged at 4 °C for 3 min. Subsequently, the supernatant was removed and 10–20 μL of 2× Laemmli sample buffer was added to each tube and thoroughly resuspend the beads. The bead samples were boiled for 2 min and cooled down in order to subject to Western blot electrophoresis.

### 4.7. F-actin/G-actin Ratio Assay

Cells were seeded at 150 mm dish and processed with Y27632 or Ang II treatment. Then, the medium was aspirated from the dish. Cells were incubated with 1500 µL of warm LAS2 and harvested by scraping thoroughly with cell scraper. Cell lysates were incubated at 37 °C for 10 min and centrifuged at 350× *g* at room temperature for 5 min to pellet unbroken cells. The supernatant was then centrifuge at 100,000× *g* at 37 °C for 1 h to pellet F-actin and leave G-actin in the supernatant. The supernatants were removed to fresh tubes designated as supernatant samples. The pellets were added into 1500 µL of F-actin depolymerization buffer and incubated on ice for 1h to allow actin depolymerization to occur. Each of the pellet and supernatant samples were added into 25 µL of 5 × SDS sample buffer and mixed well. The samples were then ready for actin quantitation by SDS-PAGE and Western blot analysis.

### 4.8. F-actin Morphology

To detect F-actin morphology, cells were seeded and attached to gelatin-coated glass coverslips. The cells were fixed with 4% paraformaldehyde in PBS for 20 min and permeabilized with 0.2% Triton X-100 for 30 min. After blocking with 5% bovine serum albumin, the cells were stained with rhodamine-conjugated phalloidin (Life Technologies) for 60 min at room temperature. After washing with PBS, the cells were stained with diamidino-2-phenylindole (DAPI) for 20 min. Stained cells were viewed under a fluorescent microscope equipped with appropriate filters (Nikon, Japan).

### 4.9. Animals and Ang II Infusion

Animal procedures were performed in accordance with the Ethics Committee and the Animal Care and Use Committee of Nantong University (Ethic Committee approval number: 1213201.1) and conformed to the NIH Guide for the Care and Use of Laboratory Animals. Male C57Bl/6 mice aged 42 days weighing 20 ± 1 g were purchased from Beijing Vital River Laboratory Animal Technology Co., Ltd. (Beijing, China). Animals were randomly assigned into the following 5 groups (*n* = 10/group): control group (normal saline infusion), model (Ang II infusion) group, Ad-RhoA-shRNA treated group, Fasudil treated group, Irbesartan treated group. For Ang II infusion, mice were implanted with an Alzet Model 1002 osmotic minipump (Alzet Corp) for subcutaneous infusion of Ang II at a rate of 1000 ng/kg/min [12]. The adenoviral vectors specific for RhoA shRNA were purchased from Hanheng Biotechnology (Shanghai, China). RhoA shRNA: GCAGATATCGAGGTGGATGGATTCAAGAGATCCATCCACCTCGATATCTGCTTTTTT. Approximately 0.1 mL virus solution (titer of 1 × 10^10^ pfu) was injected in the right common carotid artery segment (4 cm). Irbesartan (50 mg/kg/d) and fasudil (30 mg/kg/d) were added to drinking water for consumption by mice. Fourteen days later, the carotid arteries were removed, washed in ice-cold saline, and processed for subsequent analysis.

### 4.10. Masson’s Trichrome Staining

Segments of the right common carotid arteries were fixed in 4% paraformaldehyde and then embedded in OCT. The embedded sections (5-μm) were incubated overnight at room temperature in Bouin’s fixative and stained with a Masson’s Trichrome Kit. Nuclei were stained with Weigert’s hematoxylin, myofiber cytoplasms were stained with Scarlet Red, and collagen was stained with Aniline Blue dye. All images were captured using an Olympus digital camera (Olympus, Tokyo, Japan) and analyzed using Image-Pro Plus software.

### 4.11. Immunofluorescent Assay

The sections were fixed with 4% paraformaldehyde, quenched with 5% BSA blocking buffer at 37 °C for 30 min, and incubated with the mixture of two primary antibodies (rabbit anti-α-SMA and mouse anti-CD31) overnight at 4 °C. After washing three times with PBS, the sections were incubated with a mixture of the two secondary antibodies (FITC-conjugated anti-rabbit IgG and Cy3-conjugated anti-mouse IgG) for an additional 30 min at 37 °C. Staining was then visualized using a fluorescence microscope (Nikon) with the B-2A (EX: 450–490, DM: 505, BA: 520) and G-2A (EX: 510–560, DM: 575, BA: 590) filters after DAPI staining of tissues for 20 min. Fluorescence intensity was quantified using the Image-Pro Plus software program.

### 4.12. Statistical Analysis

All results are expressed as means ±SDs. One-way ANOVA followed by Tukey’s post-hoc tests were used for statistical analysis employing SPSS 22.0 software. Statistical significance was set at *p* < 0.05.
